# Heart failure diagnosis: Impacts of atrial fibrillation on the diagnostic marker NT-proBNP

**DOI:** 10.1371/journal.pmed.1004782

**Published:** 2025-10-31

**Authors:** Yang Chen, Garry McDowell, Gregory Y. H. Lip

**Affiliations:** 1 Liverpool Centre for Cardiovascular Science at University of Liverpool, Liverpool John Moores University and Liverpool Heart and Chest Hospital, Liverpool, United Kingdom; 2 Department of Cardiovascular and Metabolic Medicine, Institute of Life Course and Medical Sciences, University of Liverpool, Liverpool, United Kingdom; 3 School of Pharmacy and Biomolecular Sciences, Liverpool John Moores University, Liverpool, United Kingdom; 4 Danish Centre for Health Services Research, Department of Clinical Medicine, Aalborg University, Aalborg, Denmark; 5 Medical University of Bialystok, Bialystok, Poland

## Abstract

A recent *PLOS Medicine* study shows that atrial fibrillation lowers the specificity of the biomarker NT-proBNP for heart failure. Adjusted thresholds and better echocardiography access are therefore required for NT-proBNP to remain as a high negative predictive value rule-out test in primary care.

Heart failure (HF) and atrial fibrillation (AF) frequently coexist, engaging in a bidirectional, mutually reinforcing relationship through a shared network of structural, electrical, and neurohormonal mechanisms (Fig 1). HF predisposes to AF through chronically elevated atrial pressures, chamber dilation, interstitial fibrosis, and activation of profibrotic and proinflammatory pathways [1]. Conversely, AF can precipitate or worsen HF via tachycardia-induced cardiomyopathy, impaired ventricular filling, and secondary mitral or tricuspid regurgitation [1]. Together, these bidirectional processes create a complex and dynamic hemodynamic environment that complicates clinical evaluation (Fig 1).

**Fig 1:**
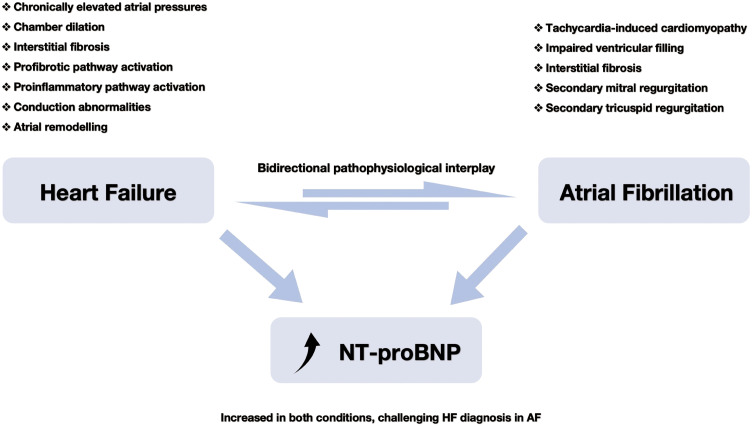
HF–AF interactions and their effects on NT-proBNP. This schematic illustrates the bidirectional interplay between HF and AF, both contributing to NT-proBNP elevation through shared and distinct mechanisms. HF promotes AF via elevated atrial pressures, chamber dilation, remodeling, fibrosis, conduction abnormalities, and inflammatory and profibrotic pathway activation. Conversely, AF worsens HF by inducing tachycardia-mediated cardiomyopathy, impaired ventricular filling, interstitial fibrosis, and secondary valvular regurgitation. These reciprocal processes drive structural and functional deterioration, collectively increasing NT-proBNP levels. As a result, interpreting NT-proBNP to diagnose HF in AF patients remains a clinical challenge. Abbreviations: AF, atrial fibrillation; HF, heart failure; NT-proBNP, N-terminal pro-B-type natriuretic peptide.

In this context, biomarkers, particularly N-terminal pro-B-type natriuretic peptide (NT-proBNP), have assumed a central role in the diagnostic pathway of patients with HF, offering some objective biochemical support to clinical judgment. However, interpretation of NT-proBNP levels in patients with associated AF is not straightforward. As a rule-out biomarker, NT-proBNP demonstrates a high negative predictive value (NPV) (typically 94%–98%), but both NPV and sensitivity/specificity are highly dependent on the diagnostic threshold applied.

In AF, NT-proBNP concentrations may be elevated through multiple mechanisms, including acute hemodynamic perturbations during arrhythmia, chronic atrial dilation and fibrosis, and increased atrial wall stretch [2], often independent of systolic function, which complicates its diagnostic use for HF. Consequently, elevated levels without overt HF reduce specificity and increase misclassification, a key concern in primary care where NT-proBNP guides further investigation.

In a recent *PLOS Medicine* study, Jones and colleagues report a large-scale, population-based cohort study evaluating the diagnostic performance of NT-proBNP for identifying HF in patients with and without preexisting AF [3]. The analysis was based on a total cohort of 155,347 individuals who underwent NT-proBNP testing in UK primary care between 2004 and 2018, linked to diagnostic records across care settings. Among these, 17,403 (11.2%) had preexisting AF. Within this broader cohort, 14,585 patients were subsequently diagnosed with HF within 6 months of NT-proBNP testing, providing a large and clinically diverse subset for focused evaluation. This nested HF cohort enabled the investigators to examine how AF status, demographic characteristics, and comorbidity profiles influenced the diagnostic performance of NT-proBNP for HF. Using real-world data from electronic health records, the study further modeled the implications of applying different NT-proBNP thresholds, standard and AF-specific, for specialist referral and case detection, highlighting trade-offs in sensitivity, specificity, and service burden.

The findings show that diagnostic discrimination for HF was substantially lower in patients with AF than in those without. At the guideline-recommended 125 pg/mL threshold, AF patients achieved extremely high sensitivity (98.8%) but very low specificity (13.2%), leading to high false-positive rates despite strong rule-out performance (NPV 97.3% versus 98.9% in non-AF). Raising the threshold to 400 pg/mL improved specificity to 35.5% in AF but remained well below the 84.9% observed in non-AF patients. Importantly, this adjusted threshold still preserved excellent rule-out capacity, achieving a sensitivity of 93.2% and NPV of 94.3% comparable to that of non-AF patients at the lower sensitivity of 92.9%. These findings are especially important for clinicians managing diagnostic uncertainty in AF, where symptoms often overlap with HF and false positives may lead to unnecessary referrals. The principal value of NT-proBNP in this setting lies in its ability to exclude disease with confidence, even when specificity is compromised.

In addition, subgroup analyses showed that NT-proBNP performance varied by age, body mass index (BMI), and sex. Discrimination was consistently higher in non-AF patients, with specificity particularly low in AF patients aged ≥65 years. In AF, BMI and sex had little effect, while in non-AF patients, higher BMI reduced sensitivity and NPV. Additionally, modeling suggested that threshold selection in AF had substantial implications for service demand: Lowering cutoffs would markedly increase specialist referrals, whereas raising them would reduce referrals but risk more missed diagnoses.

These observations highlight the limitations of applying uniform (or “one size fits all”) NT-proBNP thresholds across populations and underscores the need for AF-specific diagnostic strategies that balance sensitivity, specificity, and healthcare resource use.

Beyond the findings of this study, the results invite a broader consideration of the strengths and limitations of biomarkers in the diagnosis and risk stratification of HF. Biomarkers offer objective, reproducible, and standardized measures that can be obtained rapidly and (for some biomarkers) at relatively low cost, making them attractive tools across the spectrum of the patient care pathway, from primary care triage to acute hospital assessment. They are particularly valuable in settings where symptoms are nonspecific, such as in early-stage disease or in patients with multimorbidity, and where physical examination or imaging may be inconclusive [4]. Furthermore, biomarkers can be incorporated into risk prediction models, supporting both short- and long-term prognostication.

However, the utility of biomarkers as stand-alone diagnostic tools is constrained by their non-specificity [4]. A single biomarker may be influenced by multiple pathophysiological processes, which can complicate its diagnostic interpretation. For instance, NT-proBNP concentrations are elevated both in HF and AF. This means that while biomarkers may raise suspicion for underlying pathology, they cannot definitively confirm it without additional context or supplementary investigations. Such non-specificity also means that, for many biomarkers, their greatest clinical value lies in supporting disease exclusion rather than confirmation (i.e., “rule out” rather than “rule in”), requiring interpretation in the appropriate clinical context.

Specifically, NT-proBNP levels are modulated by diverse physiological and pathological factors, as illustrated in this study [3] by the substantial variation in diagnostic accuracy across different patient subgroups. Long-term trends in NT-proBNP with aging have been previously reported [5], likely reflecting vascular stiffening, remodeling, neurohormonal changes, and reduced renal clearance. While age must be considered in interpretation, reliance on routine health records in Jones and colleagues’ study introduces risks of confounding, misclassification, and heterogeneity due to variable test-to-diagnosis intervals [6].

While aging exerts a gradual and largely irreversible effect on NT-proBNP, cardiac rhythm has an acute and reversible impact. In persistent AF, levels are higher than in sinus rhythm, decline after cardioversion, but remain elevated with recurrence, reflecting hemodynamic and neurohormonal changes [7]. The reduced specificity in AF patients observed by Jones and colleagues [3] underscores the need to integrate biomarker results with clinical assessment, though limited echocardiographic data restrict evaluation across HF phenotypes.

Ethnicity also influences NT-proBNP, with non-Hispanic Black and Mexican American adults showing 34% and 23% lower levels, respectively, than White adults after adjustment, even in healthy groups [8]. Since Jones and colleagues’ thresholds were based on a predominantly White UK cohort, caution is needed when extrapolating to diverse populations, given known racial differences in AF, HF, and related outcomes [9–12].

The findings of this study [3] have direct implications for primary care pathways in suspected HF, especially the markedly reduced specificity of NT-proBNP in patients with AF, particularly in older adults. Relying solely on NT-proBNP risks over-referral due to nonspecific elevations. Expanding echocardiography access in primary care could reduce false positives, while in resource-limited settings, higher or context-specific thresholds combined with clinical assessment may better balance sensitivity and specificity. Ultimately, patients, not test results, should guide decisions.

Beyond these implications, the results raise an important question: should we move towards a more systematic and personalized interpretation of NT-proBNP? Rather than relying on fixed thresholds, integrated models incorporating age, AF, renal function, and other modifiers may offer a more precise probability of HF and support tailored patient care.

Overall, the major strength of the study lies in its use of a large, real-world primary care dataset that reflects contemporary practice and provides clinically relevant insights for front-line decision-making. At the same time, reliance on routinely collected health records inevitably introduces limitations, particularly residual confounding, potential misclassification, and missing data, which should be borne in mind when interpreting the findings.

## Abbreviations

AF: atrial fibrillation
BMI: body mass index
HF: Heart failure
NPV: negative predictive value
NT-proBNP: N-terminal pro-B-type natriuretic peptide

## References

[1] Newman JD, O’Meara E, Böhm M, Savarese G, Kelly PR, Vardeny O, et al. Implications of atrial fibrillation for guideline-directed therapy in patients with heart failure: JACC state-of-the-art review. J Am Coll Cardiol. 2024;83(9):932–50. doi: 10.1016/j.jacc.2023.12.033 38418008

[2] Nasab Mehrabi E, Toupchi-Khosroshahi V, Athari SS. Relationship of atrial fibrillation and N terminal pro brain natriuretic peptide in heart failure patients. ESC Heart Fail. 2023;10(6):3250–7. doi: 10.1002/ehf2.14542 37776150 PMC10682909

[3] Jones NR, Taylor KS, Ordóñez-Mena JM, Goyder CR, Hobbs FR, Taylor CJ. NT-proBNP testing for heart failure diagnosis in people with atrial fibrillation: diagnostic accuracy study. PLOS Med. 2025;22(10). 10.1371/journal.pmed.1004550 PMC12574882 41166233

[4] Esteve-Pastor MA, Roldán V, Rivera-Caravaca JM, Ramírez-Macías I, Lip GYH, Marín F. The use of biomarkers in clinical management guidelines: a critical appraisal. Thromb Haemost. 2019;119(12):1901–19. doi: 10.1055/s-0039-1696955 31499565

[5] Luchner A, Behrens G, Stritzke J, Markus M, Stark K, Peters A, et al. Long-term pattern of brain natriuretic peptide and N-terminal pro brain natriuretic peptide and its determinants in the general population: contribution of age, gender, and cardiac and extra-cardiac factors. Eur J Heart Fail. 2013;15(8):859–67. doi: 10.1093/eurjhf/hft048 23568644

[6] Song H, Ahn J-H, Kang MG, Kim K-H, Bae JS, Cho SY, et al. Post-PCI risk assessment by inflammation activity according to disease acuity and time from procedure. Thromb Haemost. 2023;123(6):627–40. doi: 10.1055/a-2011-8426 36634702

[7] Kallergis EM, Manios EG, Kanoupakis EM, Mavrakis HE, Goudis CA, Maliaraki NE, et al. Effect of sinus rhythm restoration after electrical cardioversion on apelin and brain natriuretic Peptide prohormone levels in patients with persistent atrial fibrillation. Am J Cardiol. 2010;105(1):90–4. doi: 10.1016/j.amjcard.2009.08.656 20102897

[8] Commodore-Mensah Y, Wang D, Jeon Y, Foti K, McEvoy JW, Coresh J, et al. Racial and ethnic differences in circulating N-terminal pro-brain-type natriuretic peptide (NT-proBNP) in US adults. Am J Prev Cardiol. 2023;15:100526. doi: 10.1016/j.ajpc.2023.100526 37560479 PMC10406957

[9] Kang DS, Yang PS, Kim D, Jang E, Yu HT, Kim TH, et al. Racial differences in ischemic and hemorrhagic stroke: an ecological epidemiological study. Thromb Haemost. 2024;124(9):883–92. doi: 10.1055/a-2278-8769 38423097

[10] Zakai NA, Olson NC, Judd SE, Kleindorfer DO, Kissela BM, Howard G, et al. Haemostasis biomarkers and risk of intracerebral haemorrhage in the REasons for Geographic and Racial Differences in Stroke Study. Thromb Haemost. 2017;117(9):1808–15. doi: 10.1160/TH17-03-0189 28692106 PMC6309529

[11] Allan V, Honarbakhsh S, Casas J-P, Wallace J, Hunter R, Schilling R, et al. Are cardiovascular risk factors also associated with the incidence of atrial fibrillation? A systematic review and field synopsis of 23 factors in 32 population-based cohorts of 20 million participants. Thromb Haemost. 2017;117(5):837–50. doi: 10.1160/TH16-11-0825 28229164 PMC5442605

[12] Kang DS, Yang PS, Kim D, Jang E, Yu HT, Kim TH, et al. Racial differences in bleeding risk: an ecological epidemiological study comparing Korea and United Kingdom subjects. Thromb Haemost. 2024;124(9):842–51. doi: 10.1055/a-2269-1123 38359877 PMC11349425

